# Efficacy of Sacubitril/Valsartan in the Setting of Acute Heart Failure: A Systematic Review

**DOI:** 10.7759/cureus.18740

**Published:** 2021-10-13

**Authors:** Moiud Mohyeldin, Lorena B Tavares, Mustafa Boorenie, Deya Abureesh, Saman Ejaz, Lubna Durrani, Safeera Khan

**Affiliations:** 1 Research, California Institute of Behavioral Neurosciences and Psychology, Fairfield, USA; 2 Bioethics, California Institute of Behavioral Neurosciences and Psychology, Fairfield, USA; 3 Research, California Institute of Behavioral Neurosciences & Psychology, Fairfield, USA; 4 Neurological Surgery, California Institute of Behavioral Neurosciences and Psychology, Fairfield, USA; 5 Obstetrics and Gynaecology, California Institute of Behavioural Neurosciences & Psychology, Fairfield, USA; 6 Internal Medicine, California Institute of Behavioral Neurosciences & Psychology, Fairfield, USA

**Keywords:** advanced heart failure, adhf, hfref, acute decompensated heart failure, heart failure, acute heart failure, entresto, angiotensin neprilysin inhibitor, arni, sacubitril/valsartan

## Abstract

Acute decompensated heart failure (ADHF) is one of the conditions associated with high rates of mortality and morbidity, in addition to its economic burden. Sacubitril/valsartan, the emerging drug in the field of heart failure, has been showing favorable outcomes in patients with heart failure with reduced ejection fraction (HFrEF). However, its efficacy in patients with acute decompensated heart failure remains obscure. This systematic review aims to offer more clarity to this established gap of knowledge. PubMed, ScienceDirect, and ScienceOpen were explored to gain access to studies on this topic. We conducted a systematic review to evaluate the safety and efficacy of using sacubitril/valsartan in the acute setting. Five clinical trials, 10 observational studies, including two abstracts, in addition to seven case reports and one editorial, were obtained and analyzed. Key outcomes of interest were safety and tolerability, efficacy reflected by N-terminal proB-type natriuretic peptide (NT-proBNP), and other serum and echocardiographic parameters. Additionally, target dose attainment, rehospitalization rates, and hemodynamics effect were also outcomes of interest. Based on our findings, the use of sacubitril/valsartan in patients with ADHF and cardiogenic shock is an effective measure. Although most of the results pointed to its safety, some of them showed the outcome of serious adverse events recommending its cautious use.

## Introduction and background

Acute heart failure (AHF) is defined as a worsening of symptoms and signs of heart failure (HF) demanding urgent management of the condition. It is one of the most frequently encountered diseases in medical practice, leading to high morbidity and mortality and substantial financial impact [1]. In the US, it accounts for more than one million hospitalizations per year [2]. The in-hospital mortality ranges from 4% to 7% according to the Acute Decompensated Heart Failure National Registry (ADHERE) [3], Organized Program to Initiate Lifesaving Treatment in Hospitalized Patients With Heart Failure (OPTIMIZE-HF) [4], and the EuroHeart Failure Survey (EHFS) registries [5]. However, the management of this syndrome has been a challenging entity [1].

Sacubitril/valsartan is an oral combination of angiotensin receptor blocker and neprilysin inhibitor (ARNI), based on the Prospective Comparison of ARNI with ACEI to Determine Impact on Global Mortality and Morbidity in Heart Failure (PARADIGM-HF) trial, approved in 2015 [6]. The PARADIGM-HF trial, which compared sacubitril/valsartan with enalapril in the outpatient setting, showed its superiority in comparison with enalapril in reducing the risk of the combined endpoint of cardiovascular (CV) death or hospitalization for HF [6]. The Comparison of Sacubitril/Valsartan Versus Enalapril on Effect on NT-proBNP in Patients Stabilized From an Acute Heart Failure Episode (PIONEER-HF) trial provides evidence on the efficacy of sacubitril-valsartan in contrast to enalapril in patients with acute heart failure demonstrated by a more significant reduction in the N-terminal proB-type natriuretic peptide (NT-proBNP) concentration [7]. However, the data supporting the use of sacubitril/valsartan in the acute setting remains limited [8]. Moreover, the data showing its effect in patients with cardiogenic shock remains scarce. Despite recent advances in the management of cardiogenic shock, clinical outcomes remain poor, with more than 40% mortality rates [9].

In this study, we aim to review original research publications that have evaluated the safety and efficacy of sacubitril/valsartan in the setting of acute heart failure and cardiogenic shock.

## Review

Methods

The design of this systematic review and its results used the Preferred Reporting Items for Systematic Reviews and Meta-Analyses (PRISMA) guidelines for reporting, and we adhered to its principles [10].

Search Strategy 

The following five databases were electronically scrutinized thoroughly - PubMed, PubMedCentral (PMC), Medical Literature Analysis and Retrieval System Online (MEDLINE), ScienceOpen, and ScienceDirect - using the suitable keywords and medical subject headings (MeSH) terms to precisely extract all relevant articles demonstrating the efficacy of sacubitril/valsartan in acute decompensated heart failure (ADHF). The keywords used include acute heart failure, decompensated heart failure, sacubitril valsartan, angiotensin neprilysin. We used the boolean scheme to formulate the keywords and MeSH strategy format and subsequently employed it in the databases. We subsequently retrieved all articles and carefully checked the references to avoid disregarding potentially relevant articles. Subsequently, the titles and abstracts, in addition to the subject headings were closely inspected for relevance.

Inclusion and Exclusion Criteria

We limited our choice of studies to articles published from 2011 to 2021. In addition, only articles published in the English language were included. The use of population, intervention, comparison, outcomes, and study criteria (PICOS) was the basis of selecting our eligibility criteria.

Data Extraction

Data selection and extraction were carried out separately by two researchers (first and second authors). In cases of disagreements, both authors reviewed the study design, the relevance to our inclusion and exclusion criteria, intervention used, and outcomes measured. In instances when common ground could not be achieved, we solicited the assistance of a third reviewer.

Quality Assessment of the Studies

The clinical trials were critically appraised with the Cochrane risk of bias assessment tool 2, while the systematic reviews were evaluated by the assessment of multiple systematic reviews (AMSTAR) tool. In addition, the Joanna Briggs Institute (JBI) critical appraisal checklist was used for case reports and observational studies. For other papers, the Scale for the Assessment of Narrative Review Articles (SANRA) was implemented. With the Cochrane risk of bias tool, each study was precisely chosen based on seven criteria to dig out potential biases. The score of each criterion was reported as either high quality, low quality, or unclear. Similarly, the AMSTAR was used for quality appraisal of the systematic reviews. We evaluated the essential methodological quality of the research papers through this method, with a score of eight and above being the benchmark for inclusion. In addition, using the JBI tool for observational studies and case reports, a 30% risk of bias was the maximum tolerated percentage.

Results

**Figure 1:**
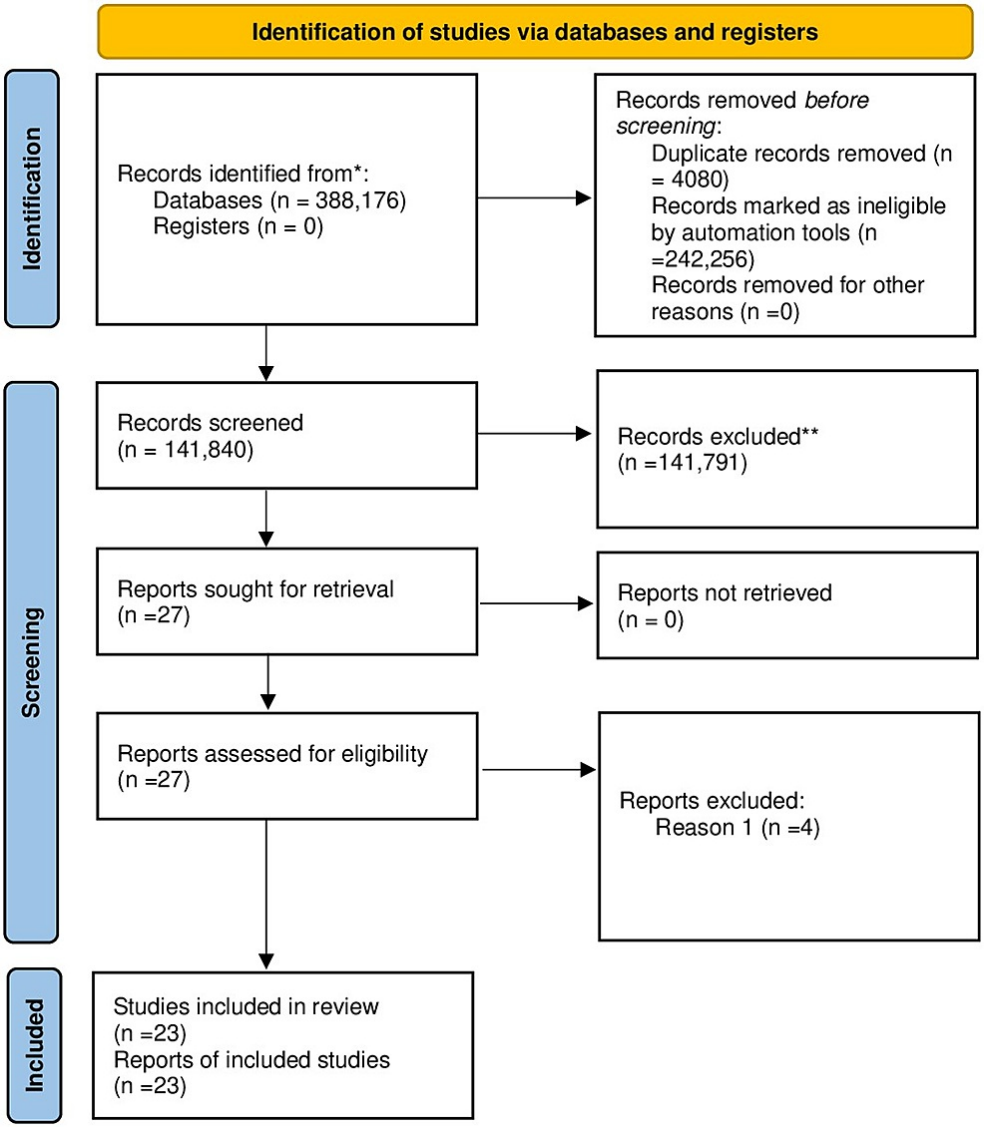
Preferred Reporting Items for Systematic Reviews and Meta-Analyses (PRISMA) flow diagram

A total of 388,176 articles were found using multiple search strategies. Out of these 388,176 articles, 1,722 articles were from PubMed, 1,102 studies from ScienceOpen, and 385,252 articles were obtained from ScienceDirect; 384,096 articles remained after the removal of 4080 duplicate articles. Based on the relevance to our ongoing research, the remaining studies were filtered by going through the titles and the content of their corresponding abstracts. Out of which, 384,018 articles were dropped due to irrelevance. Consequently, 78 articles were left, and we checked for availability of full texts, 51 articles were subsequently removed. Out of the remaining articles, 23 articles based on the eligibility criteria and following the critical appraisal were selected. Five clinical trials, 10 observational studies, including two abstracts, in addition to seven case reports and one editorial, were finalized. Shown below in Figure 1 is the complete Preferred Reporting Items for Systematic Reviews and Meta-Analyses (PRISMA) flow diagram.

The studies differed in study designs, interventions, and outcomes measured. Nevertheless, the overall goal of the studies was similar. We can see a tabulated summary below in Table 1.

**Table 1:** A tabulated summary of the studies Abbreviations: n = number of patients; S/V = Sacubitril/valsartan; HF = Heart failure; ADH = Acute decompensated heart failure; MR = Mitral regurgitation; PH =Pulmonary Hypertension; EF = Ejection fraction; LV = Left ventricle; IV = Intravenous; HFrEF = Heart failure with reduced ejection fraction; hs-TnT = High sensitivity troponin T; cTnT = Cardiac troponin T; NT-proBNP =  N-terminal pro b-type natriuretic peptide; PAPi = Pulmonary artery pulsatility index; TRANSITION trial = Study comparing pre‐discharge and post‐discharge treatment initiation with sacubitril/valsartan in heart failure patients with reduced ejectIon‐fraction hospItalised for an acute decompensation event; PIONEER-HF trial = Study comparing sacubitril/valsartan versus enalapril on effect on NT-proBNP in patients stabilized from an acute heart failure episode; ARB = Angiotensin receptor blockers; ACE = Angiotensin-converting enzyme; rhBNP = Recombinant human brain natriuretic peptide

Authors; trial name (if applicable)	Study design	Patients (n)	Intervention	Results of primary outcomes	Other results
Velazquez et al.; PIONEER-HF [7]	Double-blinded, multicentre, randomized controlled trial	n = 881	S/V (n = 440) vs enalapril (n = 441)	Time-averaged change NT-proBNP, from baseline to the mean of weeks four and eight was 47% in those taking S/V versus a 25% reduction in those taking enalapril.	S/V was safe in acute HF and new-onset HF patients. Significant reduction in HF hospitalizations.
DeVore AD et al.; PIONEER-HF [11]	Secondary analysis of the open-label extension of the PIONEER-HF trial	n = 831	Continuing S/V (n = 417) vs enalapril switching to S/V (n = 415)	Time-averaged change of NT-proBNP in the period from eight weeks to 12 weeks: For patients who continued to take sacubitril/valsartan, NT-proBNP levels declined by 17.2%. For those switching from taking enalapril to S/V, it declined by 37.4%.	Patients on S/V since hospitalization had a lower hazard for rehospitalization or cardiovascular death than patients who initiated enalapril in the hospital and then initiated S/V eight weeks later.
Velazquez et al.; PIONEER-HF [12]	Secondary analysis of the PIONEER-HF trial	n=881	S/V vs enalapril in black patients (n=316) ; white patients (n=515); others (n=50)	Among black patients admitted for ADHF, S/V resulted in a significant reduction in NT-proBNP levels.	S/V was safe and well-tolerated among black patients and significantly improved clinical outcomes compared to enalapril.
Wachter et al.; TRANSITION [13]	Open-label, multicentre, randomized controlled trial	n = 1002	Predischarge (n = 500) vs postdischarge (n = 502) initiation of S/V	Percentage of patients achieving the target dose of S/V at 10 weeks: Predischarge, 45.4%; postdischarge, 50.7%.	S/V was safe and tolerated well in acute HF and new-onset HF patients.
Senni M et al.; TRANSITION [14]	Post-hoc analysis; a subgroup analysis of the TRANSITION study	n = 991	De novo HFrEF (n=286) vs prior diagnosis of HFrEF (n=705)	The percentage of patients achieving the target dose of S/V at 10 weeks was greater in De novo HFrEF; 56%, while in prior HFrEF it was 45%.	Initiation of S/V was associated with a significant reduction in both hs-TnT and NT-proBNP in both groups.
Pang, Zhihua, et al. [15]	Open-label, single-center, randomized controlled trial	n = 300	Basic HF treatment (n=100) vs basic treatment combined with rhBNP (n=100) vs basic HF treatment execluding ACE/ARB with rhBNP followed by S/V (n = 100)	The S/V treatment group had superior outcomes in terms of cardiac structure, pulmonary artery pressure, and cardiac biomarkers (NT-proBNP Levels and cTnT levels).	S/V significantly reduced the serum levels of inflammatory factors, oxidizing factors, and increased antioxidant factors.
M Fudim et al. [16]	A retrospective observational study	n = 99767	Patients eligible for S/V using PIONEER criteria (n=20 704) vs patients eligible for S/V using actionable criteria (n = 68739)	There is a slight difference in patients' characteristics and clinical outcomes eligible for PIONEER-HF compared to those encountered in routine practice.	All-cause mortality and readmission rate were similar in both groups.
Carballo D et al. [17]	A prospective cohort study	n = 799	Patients eligible for S/V (n = 123) vs Patients non-eligible for S/V with EF<40% (n = 138) and patients non-eligible for S/V with EF>40% (n = 538)	Similar clinical outcomes (including all-cause mortality and readmission) in both eligible and non-eligible groups were noted.	
Liang HW et al. [18]	A retrospective cohort study	n = 1278	S/V	Lower risk of all-cause mortality, cardiovascular death, and HF rehospitalizations within one year.	The intervention was associated with more significant medical expenses.
Martyn et al. [19]	Retrospective observational study	n = 22	S/V	Hemodynamic improvement in ICU patients switched from vasoactive IV therapy to oral S/V therapy.	Improvement in PAPi with S/V compared to both admission and vasoactive therapy.
Martyn T et al. [20]	Retrospective observational study	n = 22	S/V	Favorable hemodynamic impact and tolerability in cardiogenic shock patients using S/V	Hypotension was the most common cause of intolerance.
Yaranov D et al. [21]	Prospective observational study	n = 10	S/V	Patients with cardiogenic shock tolerated initiation of S/V subsequent successful weaning of IV vasodilator or inotropic therapy.	
Chng BLK et al. [22]	Retrospective observational study	n = 840	Inpatient S/V (n = 89) vs Outpatient S/V (n = 551)	Initiation of S/V in the inpatient group was associated with higher ADRs and discontinuation rates than in the outpatient group.	The inpatient population tolerated S/V.
Akerman CC et al. [23]	Retrospective observational study	n = 143	S/V	Most patients were tolerant of S/V, with hypotension being the most common cause of intolerance.	Patients with newly diagnosed HF were more likely to tolerate the initiation of S/V.
Peppin et al. [24]	Retrospective observational study	n = 61	S/V	The most common cause of intolerance to S/V was hypotension.	There was an improvement in EF from baseline to ≥ 30 days post-initiation of S/V.
Acanfora D et al. [25]	Case series	n = 40	S/V	S/V was found to be effective in terms of functional capacity and cardiac biomarkers (e.g., NT-proBNP)	S/V was found to be safe.
Taghavi S et al. [26]	Case series	n = 4	S/V in inotrope-dependent HF patients.	S/V use led to discontinuation of inotrope and reducing the need for inotrope in the follow-up period.	
Gerges F et al. [27]	Case report	n = 1	S/V	Improvement of symptoms and LV function following the use of S/V	After S/V was used, a significant reduction of secondary MR severity, PH, and normalization of right ventricular function was noted.
Lo SH et al. [28]	Case report	n = 1	S/V in a child.	S/V was found to be effective in a pediatric ADH in the setting of chemotherapy-induced cardiomyopathy.	
Bell TD et al. [29]	Case report	n = 1	S/V	S/V was found to be effective and led to the discontinuation of inotropes.	
Rawal HA et al. [30]	Case report	n = 1	S/V	S/V led to cardiogenic shock in advanced HF patients.	
Almazroa L et al. [31]	Case report	n = 1	S/V	S/V was unsafe in cardiogenic shock as it led to vasoplegic shock.	
Ntalianis A et al. [32]	Editorial: Expert consensus		S/V	S/V is safe and well-tolerated and results in a more significant reduction of NT-proBNP and reduction for HF rehospitalizations.	Clinical practical strategies and action plans were recommended.

The above table looked into the key outcomes of the studies, including the safety and tolerability of sacubitril/valsartan in patients with ADHF and those with cardiogenic shock. In addition, the efficacy of the therapy and its effect on hemodynamic parameters was also noted. Moreover, some studies evaluated the rates of rehospitalization and drug discontinuation during and following discharge. The sum of those studies included more than a hundred thousand patients. Most of the study results point towards a favorable outcome regarding both the safety and efficacy of the medication. NT-proBNP, functional improvement, and decreased rehospitalization rates were observed in most of the studies assessing the efficacy. By measuring the side effects and discontinuation rates of the medication, the medication was found to be safe in most of the studies.

Discussion

Safety and Tolerability

The safety and tolerability of sacubitril/valsartan were assessed in many different studies. The main outcomes evaluated included achieving the target dose, safely discharging patients on the medication, and the incidence rates of patients tolerating sacubitril/valsartan. These variables were assessed in primary or secondary outcomes, and many studies presented information on multiple outcomes.

The TRANSITION trial evaluated the safety and tolerability of initiating sacubitril/valsartan predischarge vs. postdischarge [13]. On admission for acute heart failure, patients were separated into three different groups: one on an angiotensin-converting enzyme (ACE) inhibitor, the second on an angiotensin receptor blocker (ARB), and the third on an ACE inhibitor/ARB naïve. Patients were randomized within each group to start sacubitril/valsartan either predischarge (n = 500) or postdischarge (n = 502). The primary endpoint was the number of patients achieving the target dose by the end of week 10 following randomization. The proportion of drug discontinuation due to adverse events during the 10 weeks was a key safety outcome [13]. Results of the trial at week 10 showed no significant difference between the different initiation approaches. No significant difference was observed between groups in regards to permanent discontinuation due to an adverse event. In addition, hypotension rates were higher in the predischarge group; the authors attributed this to the higher vulnerability of this group because they were initiated while inpatient post-ADHF, compared with patients initiated postdischarge [13]. In contrast to the PIONEER-HF trial [7], which did not show variables related to the incidence of the adverse events, the TRANSITION trial showed that certain baseline characteristics were associated with target dose achievement. This trial was limited by its open-label design but reinforced by its large sample size.

In a subgroup analysis of the TRANSITION trial, Senni M et al. assessed the tolerability of initiating sacubitril/valsartan following ADHF with a de novo (newly diagnosed) vs prior diagnosis of heart failure with reduced ejection fraction (HFrEF) [14]. Two hundred and eighty-six patients with de novo HFrEF and 705 with prior HFrEF were included in the study. The authors noted that more patients achieved the target dose at 10 weeks in the de novo HFrEF group (56% n = 160), and fewer had serious adverse events. In addition, they concluded that the initiation of sacubitril/valsartan was associated with a significant reduction in both high sensitivity troponin T (hs-TnT) and NT-proBNP in both groups. However, it was faster and greater in the de novo group, which reflects the efficacy of the medication.

Akerman CC et al. conducted a retrospective single-center study [23]. The main focus of the study was to assess the risk factors for intolerance of inpatient sacubitril/valsartan initiation. Those prescribed sacubitril/valsartan during the inpatient stay but were not discharged on the medication were considered intolerant. Of the 143 patients meeting entry criteria, 64.3% (n = 92) had a diagnosis of ADHF. However, of those only 20% (n = 18) were considered intolerant. In comparison to those who were tolerant of sacubitril/valsartan, the intolerant group had a higher incidence of hypotension, confirming the findings of the TRANSITION trial. Some of the limitations of this study included its small sample size, risk of selection bias, being a retrospective study, and lack of information regarding the doses received during hospitalization or at discharge. Looking more into the risk factors of intolerance, Peppin KL et al. performed a retrospective study in a community hospital to evaluate the safety and tolerability of sacubitril/valsartan in 59 patients with ADHF [24]. In this study, the intolerance was assessed on those initiating sacubitril/valsartan at least 24 hours before discharge. The primary outcome was the incidence of hypotension, which 35.6% (n = 21) of patients had suffered from. Those without a prior diagnosis of hypertension had more hypotensive events than those with a prior hypertension diagnosis. The majority (86.4%, n = 51) of patients were discharged on sacubitril/valsartan. However, only 3.9% (n = 2) of those achieved the target dose. One of the limitations of this study is the small sample size. In addition, the fact that several patients were initiated on the therapy 24 hours before discharge might have led to missing some of the adverse events. Also, sampling bias is a concern in this study.

To assess the validity of the TRANSITION and PIONEER trials in the Asian population, Chng BLK et al. conducted a retrospective observational study on 840 patients [22]. The study compared initiation of sacubitril/valsartan in inpatient (n = 289) and outpatient settings (n = 551). The study results showed that the initiation of sacubitril/valsartan in the inpatient group was associated with higher adverse drug reactions (ADRs) and discontinuation rates than in the outpatient group; however, sacubitril/valsartan was tolerated by the inpatient population as well. Being a single-center study would question the validity of this study regarding its representation of the Asian population.

A retrospective cohort study conducted by Liang HW et al. on 1278 patients with ADHF, aimed to assess the medical costs and clinical effectiveness of sacubitril/valsartan [18]. By comparing 426 patients using sacubitril/valsartan with 852 patients who did not use it, the author concluded that the intervention was associated with more significant medical expenses. However, a lower risk of all-cause mortality, cardiovascular death, and HF rehospitalizations within one year was also noted. The possibility of cofounders and the sample size are possible limitations of this study.

Acanfora D et al., in a prospective case series study including 40 patients with ADHF, assessed the safety and efficacy of sacubitril/valsartan [25]. The authors evaluated the efficacy by physical measures and serum biomarkers, while the safety was measured by renal function, hyperkalemia, and symptomatic hypotension. The study results showed that in terms of functional capacity and biomarkers, sacubitril/valsartan was safe and effective.

Efficacy

The PIONEER-HF trial was a double-blinded multi-center randomized controlled trial (RCT) that evaluated the efficacy and safety of sacubitril/valsartan treatment in hospitalized patients for ADHF [7]. A total of 881 patients with decompensated HF after fulfilling the inclusion criteria were randomly assigned to receive sacubitril/valsartan or enalapril treatment. The time-averaged change in the NT-proBNP level was the primary endpoint of the study. A greater reduction in the NT-proBNP and hs-TnT was found in the sacubitril/valsartan group compared to the enalapril group [7]. Moreover, in an analysis of the clinical outcomes, sacubitril/valsartan reduced the risk of rehospitalization for HF, but not death, reflecting the effectiveness of the therapy. In addition, there was no significant difference between the two groups regarding the rates of worsening renal function, hyperkalemia, symptomatic hypotension, and angioedema [7].

Velazquez EJ et al., on a secondary analysis of the PIONEER-HF Trial, evaluated the safety efficacy of sacubitril/valsartan on different ethnicities [12]. The analysis compared sacubitril/valsartan vs enalapril in black patients (n = 316); white patients (n = 515); others (n = 50). Among black patients, sacubitril/valsartan resulted in a significant reduction in NT-proBNP levels. In addition, it was safe and well-tolerated among the same patient population. This study shows the value of using sacubitril/valsartan regardless of the target ethnic groups.

On a secondary analysis of a four-week open-label extension of the PIONEER-HF Trial, DeVore AD et al. gave more evidence regarding the efficacy of sacubitril/valsartan [11]. This study showed that switching patients' treatment from enalapril to sacubitril/valsartan at eight weeks following randomization resulted in a further decrease in NT-proBNP levels. However, a lower hazard of composite outcome was found on those that began taking sacubitril/valsartan in the hospital compared with patients that initiated enalapril in the hospital and then eight weeks later had a delayed introduction to sacubitril/valsartan. As this study was only for four weeks, various results may have been noted with longer follow-up, reflecting a limitation of this study.

In a retrospective study using real-world data of 99767 patients, Fudim M et al. evaluated the representativeness of the PIONEER-HF trial among patients hospitalized for ADHF [16]. The study included patients eligible for sacubitril/valsartan using PIONEER criteria (n = 20704) compared with patients eligible for sacubitril/valsartan using actionable criteria (n = 68739). The study concluded that there was a minimal difference in patients' characteristics and clinical outcomes eligible for PIONEER-HF compared to those encountered in the study (reflecting routine practice). In addition, all-cause mortality and readmission rate were similar in both groups. This emphasizes the value of sacubitril/valsartan use in the real world. Having some surrogate criteria as a replacement of the PIONEER criteria might have limited this study.

Carballo D et al. conducted a prospective cohort study including 799 patients [17]. The study's main goal was to evaluate eligibility for sacubitril-valsartan using criteria mentioned in the PIONEER-HF trial in non-selected patients hospitalized for ADHF. The study divided the patient population into three groups; patients eligible for sacubitril/valsartan (n = 123) vs patients non-eligible for sacubitril/valsartan with EF<40% (n = 138) and patients non-eligible for sacubitril/valsartan with EF>40% (n = 538). Further increasing the validity of this therapy across different groups, similar clinical outcomes (including all-cause mortality and readmission rate) in both eligible and non-eligible groups were observed in this study.

Pang Z et al. conducted an open-label single-center randomized controlled trial [15]. The study included 300 patients stratified into basic HF treatment (n = 100) vs basic treatment combined with rhBNP (n = 100) vs basic HF treatment excluding ACE/ARB with rhBNP followed by sacubitril/valsartan (n = 100). The authors concluded that the sacubitril/valsartan treatment group had superior outcomes in terms of cardiac structure, pulmonary artery pressure, and cardiac biomarkers (NT-proBNP levels and cardiac troponin test [cTnT] levels). In addition, a significant reduction in serum levels of inflammatory and oxidizing factors and an increase in antioxidant factors was noted. The small sample size and it being a single-center study are possible limitations of this trial.

In a case reported by Gerges F et al., sacubitril/valsartan led to a dramatic improvement in the echocardiographic parameters and symptoms of a patient with ADHF [27]. The initiation of the therapy in the acute setting led to improvement of symptoms and left ventricular function. Moreover, a significant reduction of secondary mitral regurgitation severity and normalization of right ventricular function was noted.

Role in Cardiogenic Shock and Intensive Care Unit Setting

The data describing the use of sacubitril/valsartan in critically ill patients with cardiogenic shock remains limited. In this section, we discussed some of the observational studies and reported cases in this area.

Martyn et al., in a small retrospective study, evaluated the safety and tolerability of sacubitril/valsartan in ADHF patients with cardiogenic shock after vasoactive IV therapy [19]. This study included 22 patients who were initiated on sacubitril/valsartan in the cardiac intensive care unit (CICU). The main goals of this study were to evaluate the tolerability of sacubitril/valsartan at discharge, reasons for discontinuation, and adverse events. Patients newly initiated on sacubitril/valsartan in the CICU were managed with vasoactive IV therapy. At the time of angiotensin receptor neprilysin inhibitor (ARNI) initiation, 80% (n = 20) of patients were on either sodium nitroprusside, milrinone, dobutamine, or a combination of these agents. The authors reported that at discharge, a total of 16 patients continued treatment with sacubitril/valsartan [19]. Improved hemodynamics (e.g., decreased cardiac filling pressures, increased cardiac index) was noted in all 16 patients, and after one month, most of them (94%) remained on therapy. The key limitations of this study are the small sample size and the retrospective design.

In a similar study, Martyn T et al. conducted a single-center retrospective study in 22 patients with ADHF and cardiogenic shock admitted to the CICU, and were newly initiated on sacubitril/valsartan [20]. The main goal of the study was to evaluate the tolerability and hemodynamic impact of sacubitril/valsartan. The study showed favorable hemodynamic impact and tolerability in cardiogenic shock patients using sacubitril/valsartan. However, in those who were intolerant of the therapy, hypotension was the most common cause of intolerance. Being a retrospective study with a small sample size is a limitation of this study.

Further emphasizing the safety and efficacy of sacubitril/valsartan in the intensive care unit (ICU) setting, a prospective observational study conducted by Yarnov DM et al. on 10 patients with ADHF and cardiogenic shock in the ICU showed favorable results [21]. The study concluded that patients with cardiogenic shock tolerated the initiation of sacubitril/valsartan, and subsequent successful weaning of IV vasodilator or inotropic therapy was also noted.

In a case series of four patients with cardiogenic shock dependent on inotropes, Taghavi S et al. evaluated the use of sacubitril/valsartan [26]. The study concluded that the use of sacubitril/valsartan led to the discontinuation of inotrope and reduced the need for inotrope in the follow-up period, reflecting its efficacy. In a similar study, Bell TD et al. reported a case of a refractory advanced heart failure patient who was admitted to the ICU and started on inotropic therapy [29]. Following the use of sacubitril/valsartan, the patient's condition improved and discontinued inotropes.

However, in two different case reports reported by Rawal HA et al. and Almanzora L et al., the use of sacubitril/valsartan resulted in serious adverse events [30,31]. In the first case reported by Rawal HA et al., sacubitril/valsartan in a patient with advanced heart failure led to a cardiogenic shock. In the other case reported by Almazaro L et al., a patient with cardiogenic shock was started on sacubitril/valsartan, which resulted in vasoplegic shock. While the value of sacubitril/valsartan in the pediatric population remains unknown, Lo SH et al. reported a case of a pediatric patient with ADHF in the setting of chemotherapy-induced cardiomyopathy [28]. Following the initiation of low dose sacubitril/valsartan, the patient's condition improved, successfully reversing the acute decompensation state. The generalizability of these results is limited by being a single case study but is a promising result for further studies exploring these outcomes.

In one of the latest expert consensuses, Ntalianis A et al. described the role of sacubitril/valsartan in patients with ADHF [32]. The study concluded that sacubitril/valsartan is safe and well-tolerated and results in a significant reduction of NT-proBNP and reduction for HF rehospitalizations. In addition, the authors recommended practical clinical strategies and action plans for the implementation of the medication.

Limitations

Although this systematic review shows promising results regarding the safety and efficacy of sacubitril/valsartan in patients with ADHF, in addition to those with cardiogenic shock, this study encountered certain limitations. Certain factors may have played a role in limiting this. These include true quality of the primary studies involved, heterogeneity of inclusion criteria, and the study included only papers published in the English language from 2011 to 2021.

## Conclusions

ADHF patients are at risk for increased mortality and rehospitalizations. The outcomes of the presented studies encourage the early initiation of sacubitril/valsartan treatment in the acute setting. Based on the evidence evaluating key surrogate and clinical outcomes, initiating sacubitril/valsartan for patients with ADHF is a reasonable recommendation. Management of clinically stabilized patients hospitalized for ADHF with sacubitril/valsartan significantly reduces the risk of serious clinical events and reduces NT-proBNP concentrations. Moreover, this finding was also found in patients who are critically ill and those on inotropic therapy, but further studies would help confirm these findings. Remarkably, sacubitril/valsartan has a good safety profile. More randomized controlled trials are recommended as they would be valuable for uncovering this medication's benefits in the acute setting and critically ill patients.
